# Impacts of Drug Interactions on Pharmacokinetics and the Brain Transporters: A Recent Review of Natural Compound-Drug Interactions in Brain Disorders

**DOI:** 10.3390/ijms22041809

**Published:** 2021-02-11

**Authors:** Bikram Khadka, Jae-Young Lee, Eui Kyun Park, Ki-Taek Kim, Jong-Sup Bae

**Affiliations:** 1 Department of Biomedicine, Health & Life Convergence Sciences, BK21 Four, Mokpo National University, Muan-gun, Jeonnam 58554, Korea; khadkabikram180@gmail.com; 2 College of Pharmacy, Chungnam National University, Daejeon 34134, Korea; jaeyoung@cnu.ac.kr; 3 Department of Pathology and Regenerative Medicine, School of Dentistry, Kyungpook National University, Daegu 41940, Korea; epark@knu.ac.kr; 4 College of Pharmacy and Natural Medicine Research Institute, Mokpo National University, Muan-gun, Jeonnam 58554, Korea; 5 College of Pharmacy, Research Institute of Pharmaceutical Sciences, Kyungpook National University, Daegu 41566, Korea

**Keywords:** natural compound–drug interactions (NDIs), pharmacokinetics, drug transporters, blood–brain barrier (BBB) and blood–cerebrospinal fluid barrier (BCSFB), brain disorders

## Abstract

Natural compounds such as herbal medicines and/or phyto-compounds from foods, have frequently been used to exert synergistic therapeutic effects with anti-brain disorder drugs, supplement the effects of nutrients, and boost the immune system. However, co-administration of natural compounds with the drugs can cause synergistic toxicity or impeditive drug interactions due to changes in pharmacokinetic properties (e.g., absorption, metabolism, and excretion) and various drug transporters, particularly brain transporters. In this review, natural compound–drug interactions (NDIs), which can occur during the treatment of brain disorders, are emphasized from the perspective of pharmacokinetics and cellular transport. In addition, the challenges emanating from NDIs and recent approaches are discussed.

## 1. Introduction

Currently, more than one billion individuals worldwide suffer from brain disorders, including neurodegenerative diseases (e.g., Alzheimer’s disease and Parkinson’s disease), ischemic strokes, brain cancers, epilepsy, and traumatic brain injuries. By 2020, the aforementioned conditions constituted 14.7% of all disorders worldwide [1]. Various therapeutic agents for brain disorders have been developed, and a few of them that received United States Food and Drug Administration (FDA) approval, have been used clinically for the treatment. In addition, natural compounds such as phyto-compounds, which are derived from plants, vegetables, and fruits, and dietary nutrients have frequently been used to exert therapeutic effects owing to their own antioxidant, anti-inflammatory, and neuroprotective properties [2,3]. In patients with brain disorders, combinatorial therapy or co-administration of therapeutic drugs and natural compounds has been practiced frequently owing to synergistic therapeutic effects, multi-targeting effects, immune-boosting effects, preventive effects against other chronic diseases, and safety profiles of natural compounds [4,5]. 

However, when anti-brain disorder agents, herbal medicines, and/or other natural compounds are co-administered, harmful/impeditive drug interactions may occur owing to the changes in pharmacokinetic properties (e.g., absorption, distribution, metabolism, and excretion; ADME) and various drug transporters, particularly brain transporters [4,6]. These ADME-mediated natural compound–drug interactions (NDIs) can increase or decrease the systemic exposure of therapeutic drugs in plasma or brain thereby accelerating their therapeutic effects, occurring drug toxicity, or diminishing drug efficacy [6]. Induction or inhibition of drug transporters and metabolism enzymes by natural compounds can also lead to significant changes in drug exposure [7]. Moreover, membrane barriers like the blood–brain barrier (BBB) and the blood–cerebrospinal fluid barrier (BCSFB), which affect drug distribution into the brain, can be changed depending on disease state, and various drug transporters in the barriers can be significantly upregulated or downregulated by some natural compounds [8,9]. Therefore, understanding various physiological and biopharmaceutical factors of NDIs in the brain should be a prerequisite for the estimation and prediction of possible NDIs in brain disorders and should be addressed by the clinician. However, there is a lack of data on the pharmacokinetics of natural compounds and NDIs as well as a poor understanding of the expression and function of human brain transporters [4,10]. In this review, pharmacokinetic properties, including ADME, physical barriers such as the BBB and BCSFB, and various drug transporters that can affect drug delivery into the brain are discussed. In addition, possible NDIs, which can occur during the treatment of brain disorders, are emphasized from the perspective of pharmacokinetics and brain transporters. Moreover, the limitations with regard to estimating and predicting NDIs are summarized. 

## 2. Physiological and Biopharmaceutical Factors in the Brain 

To fully understand the possible mechanisms underlying NDIs, the effects of physiological factors, such as the BBB and BCSFB, and biopharmaceutical factors, such as ADME and drug transporters, on drug delivery into the brain should be addressed in detail. These factors can be altered by the progression of various brain diseases.

### 2.1. Physiological Barriers

**Figure 1:**
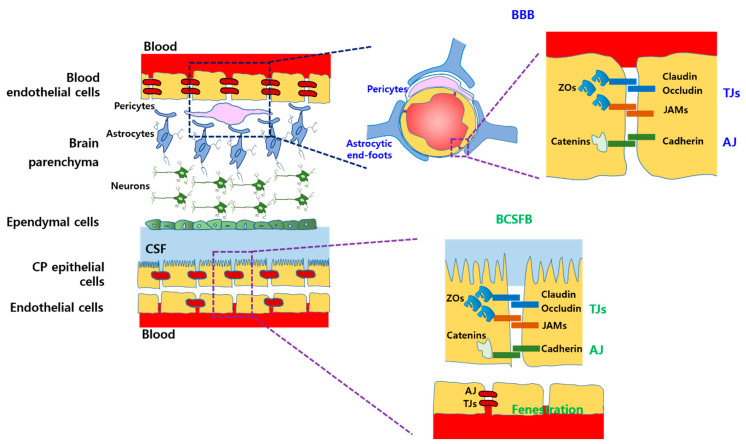
Schematic diagram of the structure of the blood–brain barrier (BBB) and blood–cerebrospinal fluid barrier (BCSFB) regarding tight junction (TJ) molecules, adherence junction (AJ) molecules, astrocytes, and pericytes.

A schematic diagram of the structure of the BBB and BCSFB is depicted in Figure 1. Two major barriers, the BBB and BCSFB, separate the brain parenchyma or brain interstitial fluid (ISF) from the blood and cerebrospinal fluid (CSF) [11]. These barriers prevent paracellular diffusion and penetration of hydrophilic entities and macromolecules, thereby maintaining homeostatic and stable brain microenvironments, mainly composed of neuronal cells [1,11]. The major barrier characteristics of the BBB and BCSFB are a result of the continuous endothelial cells and choroid plexus (CP) epithelial cells, respectively, which are interconnected with highly expressed tight junction (TJ) and adherence junction (AJ) molecules [12]. TJs on the luminal side are composed of claudin, occludin, junctional adhesion molecules (JAMs), and zonula occludens (ZOs), while AJs on the abluminal side consist of cadherin and catenins (Figure 1) [13]. As a consequence, essential nutrients such as glucose and amino acids, neurotransmitters such as dopamine and acetylcholine, and ions cannot diffuse through or penetrate the brain parenchyma. Therefore, various drug transporters and carriers that can actively transport those nutrients, neurotransmitters, and ions into the brain are expressed in the BBB and BCSFB [13,14]. In addition, efflux transporters are highly expressed in both barriers, resulting in the removal of xenobiotics, drugs, and waste molecules from the brain ISF [11,13]. Moreover, some enzymes such as esterases, aminopeptidases, and microsomal cytochrome P450 (CYP) are also expressed in both barriers, thereby contributing to metabolic hindrance in the brain [15].

Given the differences in the BBB and BCSFB, astrocytes, pericytes, and microglial cells, which cover blood capillaries of the BBB, affect the maintenance of the barrier function and support the structural integrity of the interconnected endothelial cells. In contrast, fenestration is frequently observed on endothelial cells of choroidal blood capillaries in the BCSFB without astrocytes and microglial cells, thereby allowing some molecules to cross the BCSFB (CP epithelial cells) (Figure 1). In addition, the expression and position of drug transporters in both barriers are different. 

### 2.2. Various Drug Transporters in the Brain

#### 2.2.1. Solute Carrier (SLC) Transporters

**Figure 2:**
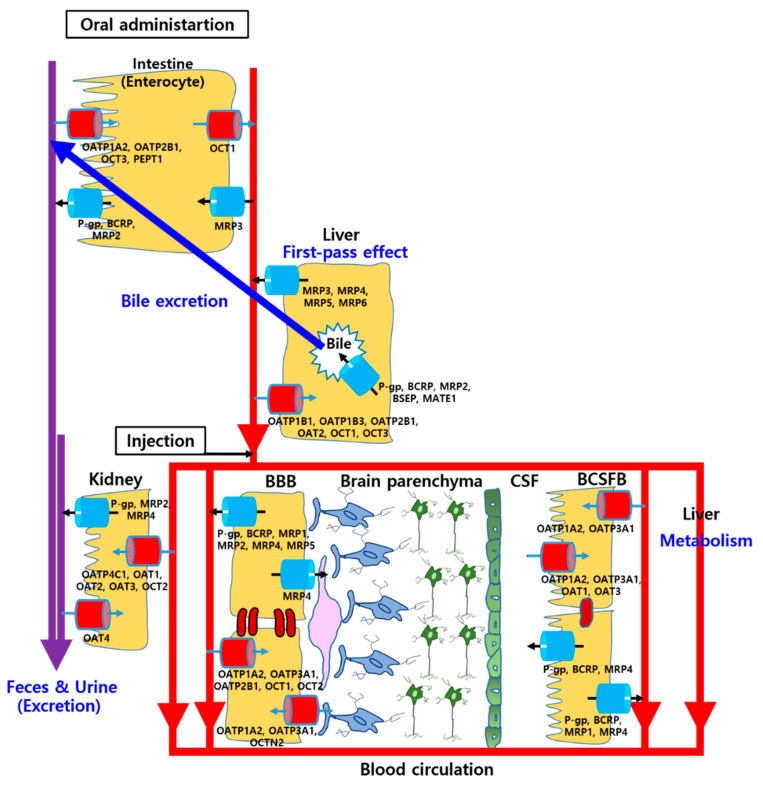
Various drug transporters on absorption, distribution, metabolism, and excretion (ADME) process and the brain after oral administration or injection.

The SLC families that do not need adenosine triphosphate (ATP) include ion-coupled transporters, exchangers, and facilitated carriers primarily involved in the influx of xenobiotics into the endothelial and CP epithelial cells [16]. Among these family, organic anion transporting polypeptides (OATPs), organic anion transporters (OATs), organic cation transporters (OCTs), l-amino acid transporters (LATs), peptide transporters (PEPTs), and glucose transporters (GLUTs) have been determined in the brain. A schematic diagram of the position of various SLC transporters in the BBB and BCSFB is depicted in Figure 2. Particularly, in humans, OATP2B1, OCT1, and OCT2 are localized at the luminal side and novel organic cation transporter 2 (OCTN2) is localized at the basolateral side of endothelial cells in the BBB, whereas OAT1 and OAT3 are positioned at the luminal side of CP epithelial cells in the BCSFB [16]. OATP1A2 and OATP3A1 are expressed at both sides in the BBB and BCSFB, respectively [17]. LAT1 and GLUT1 are localized at both sides in both BBB and BCSFB [11]. However, the exact expression and localization of SLC transporters in the human brain are not fully identified.

#### 2.2.2. ATP-Binding Cassette (ABC) Transporters

ABC transporters which are coupled with Na^+^/K^+^-ATPase pump mainly include P-glycoprotein (P-gp; ABCB1), multidrug resistance-associated proteins (MRPs; ABCC), and breast cancer resistance protein (BCRP; ABCG2) primarily involved in the efflux of xenobiotics out of the endothelial and CP epithelial cells against their concentration gradient, thereby prevention of the brain parenchyma and ISF from those molecules [16]. A schematic diagram of the position of various ABC transporters in the BBB and BCSFB is depicted in Figure 2. Particularly, in humans, P-gp and BCRP are predominantly localized at the luminal side of endothelial and CP epithelial cells in the BBB and BCSFB. MRP1 is expressed at the luminal side of blood endothelial cells, whereas, in CP epithelial cells, it is localized at the basolateral side [16,17]. MRP2 and MRP5 are expressed at the luminal side of blood endothelial cells [16]. MRP4 is positioned at both side in the both BBB and BCSFB [17]. However, the exact expression and localization of ABC transporters in human brain are not fully identified, neither. 

### 2.3. Pharmacokinetic and Pharmacodynamic Factors

#### 2.3.1. Gastrointestinal (GI) Absorption

Several factors can affect GI absorption of drugs such as gastric pH, gastric emptying time, GI transit time, and drug transports in the intestinal membrane [18]. The pH of gastric media can change solubility, dissolution, and membrane-permeation of drugs. If the drug is unstable at certain pH or the absorption site of the drug is limitative and site-specific, the gastric emptying time and GI transit time can play important roles in GI absorption of the drug. Moreover, SLC and ABC transporters are expressed at the luminal and basolateral side of enterocytes, thereby facilitating influx or efflux transportation of drugs [19]. Therefore, raising systemic exposure of drugs for brain disorders by inhibiting intestinal efflux transportation can cause enhanced blood concentration and drug delivery into the brain. A schematic diagram of the position of various transporters in the enterocytes is depicted in Figure 2. At the luminal side, OATP1A2, OATP2B1, and PEPT1 as SLC transporters, and P-gp, BCRP, and MRP2 as ABC transporters are localized, whereas OCT1 and MRP3 are expressed at the basolateral side of enterocytes [20]. 

#### 2.3.2. Distribution

In the distribution process of drugs to overall the body after the absorption, plasma protein binding can mainly influence a portion of drugs reaching target tissues or eliminative organs like the liver and kidney [21]. In the blood, human serum albumin, α-1 acid glycoprotein, and lipoprotein play an important role as the plasma proteins, thereby only dissociated portion of drugs (free drugs) can exert their own pharmacological activities [22]. Therefore, raising an unbound fraction of drugs for brain disorders, which generally means the volume of distribution is getting lower, may cause enhanced blood concentration, drug delivery into the brain, and efficacy. This interaction may occur extensively in the case of drugs with higher plasma protein binding (>95%) and narrow therapeutic indexes such as anti-epilepsy agents and warfarin [6,22]. 

#### 2.3.3. Hepatic Metabolism and Bile Excretion

Drug metabolism mediated by phase I metabolic enzymes (CYP family) and phase II metabolic enzymes (e.g., glucuronosyltransferase, *N*-acetyl transferase, glutathione *S*-transferase, and sulfonyl transferase) plays an important role in the elimination of drugs, thereby affecting their pharmacokinetic properties [23]. Those metabolic enzymes are predominantly expressed in the endoplasmic reticulum of the liver. Drug interactions during the metabolic process can mainly occur inhibition or induction of those enzymes by other CYP substrates [22]. Most drugs are metabolized by the CYP3A4, CYP1A2, CYP2C9, CYP2D6, CYP2E1, and CYP2C19, and CYP3A4 is responsible for approximately 50% of the entire drug metabolism [24]. In addition, those CYP enzymes are expressed in other hepatic tissues like enterocytes of the intestine and even whole brain tissues and the BBB. In the human intestine, CYP3A4, CYP2C9, CYP2C19, and CYP2D6 are expressed, and CYP3A4 occupies up to 80% of the entire intestinal CYP enzymes [25,26]. In human brain tissues and the BBB, CYP1A1, CYP1A2, CYP2B6, CYP2C9, CYP2C19, CYP2D6, CYP2E1, CYP3A4, and glucuronosyltransferase are expressed [27]. 

Moreover, SLC and ABC transporters are expressed on the sinusoidal membrane and canalicular membrane of hepatocytes, thereby facilitating the uptake of drugs into hepatocytes and their excretion through the bile duct [19]. Bile excretion into the duodenum of the intestine can play a key role in the elimination of drugs through feces and drug reabsorption into the intestinal membrane, called enterohepatic circulation [28]. A schematic diagram of the position of various transporters in the hepatocytes is depicted in Figure 2. At the sinusoidal side, OATP1B1, OATP1B3, OATP2B1, OAT2, OCT1, and OCT3 as SLC transporters, and MRP3, MRP4, MRP5, and MRP6 as ABC transporters are localized, whereas P-gp, BCRP, MRP2, bile salt export pump (BSEP; ABCB11), and multidrug and toxin extrusion transporter 1 (MATE1) are expressed at the canalicular side [19,29]. 

#### 2.3.4. Renal Excretion

The major route of drug excretion is renal excretion through the kidney involved with glomerular filtration, tubular reabsorption, and tubular secretion. Glomerular filtration is entirely by the renal functions and tubular reabsorption is mediated by simple diffusion with drug concentration gradients, whereas tubular secretion is mediated by drug transporters including facilitated carriers and active transporters [30]. Various SLC and ABC transporters are expressed on the luminal and basolateral side of proximal tubule cells in the kidney, thereby facilitating the uptake of drugs into the cells and their excretion through urine [31]. A schematic diagram of the position of various transporters in the renal proximal tubule cells is depicted in Figure 2. At the luminal side, OAT4 as SLC transporters, and P-gp, MRP2, and MRP4 as ABC transporters are localized, whereas OATP4C1, OAT1, OAT2, OAT3, and OCT2 are expressed at the basolateral side [19,31]. 

Therefore, raising systemic exposure of drugs for brain disorders by inhibiting hepatic metabolism, bile or renal excretion can cause enhanced blood concentration and drug delivery into the brain. 

#### 2.3.5. Pharmacodynamic Synergy, Addition, and Antagonism

Pharmacodynamic drug interactions can be caused when drugs bind to the same target receptors or the different receptors that have similar or opposite activities, thereby the pharmacological effects of drugs can be affected by each other [32]. Particularly, since one natural compound can have multiple targets for its pharmacological activities and mixtures of natural compounds like the extracts have diverse constituents, pharmacodynamics NDIs may occur considerably [33,34]. Pharmacodynamic drug interactions are sub-categorized as synergism, addition, and antagonism. Additive effects can occur when the drugs have no interaction with each other, resulting in just a summation of that efficacy. The exact molecular mechanisms of drug synergism or antagonism are not fully understood, but some models based on Loewe’s and Bliss’s definition can be used to evaluate and predict these interactions [34,35]. 

### 2.4. Changes of Physiological and Biopharmaceutical Factors in Brain Disorders

Considering pharmacokinetic properties of drugs, especially their distribution into the brain, can be affected by the disease state of patients with brain disorders, NDIs in brain disorders may occur more severely compared to in normal conditions [36]. Therefore, understanding the changes of physiological and biopharmaceutical factors in brain disorders is preceded to identify and predict possible NDIs in the patients with those diseases. The changes in brain disorders are mainly related to various drug transporters expressed in the BBB and BCSFB and these barrier functions. Previous studies reported that brain disorders, such as multiple sclerosis, dementia, stroke, and brain cancer, or even, aging can cause disruption of TJs and AJs, resulting in the leaky BBB and BCSFB [36,37,38]. In addition, the expression of ABC transporters (e.g., P-gp, BCRP, and MRPs) as drug efflux pumps can be upregulated in the BBB and BCSFB of patients with brain cancer [39]. Moreover, those ABC transporters are overexpressed in the BBB of epileptic patients, leading to cause drug resistance of various anti-epileptic agents [40]. In ischemic stroke models, the enhanced expression of P-gp was also observed, thereby impeding drug delivery into the damaged brain [41]. However, during Alzheimer’s disease (AD), the expression of P-gp, BCRP, and lipoprotein receptor-related protein 1 in the BBB is downregulated, resulting in reducing clearance of amyloid plaque and enhancing its accumulation in the brain tissues [42,43]. Moreover, the reduced expression of GLUT1 was observed due to decreased need for glucose in the damaged brain tissues [43]. In patients with Parkinson’s disease, the reduced expression of P-gp and dysfunction of P-gp and BCRP in the BBB have been reported [43,44]. In addition, the expression of LAT1 can be downregulated, resulting in the reduction of dopamine or levodopa uptake into the brain [45]. 

## 3. Natural Compound–Drug Interactions in Brain Disorders

### 3.1. Possible NDIs in Clinical Usage for Brain Disorders

Several clinical studies have reported that natural compounds that have been commonly intake can affect oral availability, systemic exposure, and/or hepatic clearance of co-administered drugs for brain disorders with different mechanisms [46]. Combination of natural compounds and various drugs for brain disorders causing NDIs in clinical was summarized in Table 1.

Carbamazepine (CBZ), widely used as conventional antiepileptic drugs, is known as a substrate of CYP3A4 and potential CYP enzyme inducer. For CBZ, NDIs can play a key role in exhibiting its efficacy and/or side effects, thus the related NDI researches in clinical have been assessed widely [47]. Intake of grapefruit juice or orange juice can inhibit CYP3A4-mediated metabolism, thereby enhancing the oral bioavailability and systemic exposure of CBZ in patients with epilepsy [48,49]. Resveratrol and diosmin can also enhance systemic exposure of CBZ and prolong its blood circulation by inhibiting CYP3A4-mediated metabolism [50,51]. Piperine, which is commonly contained in the daily Indian diet from black pepper and dried rhizomes of ginger, can enhance oral bioavailability and systemic exposure of CBZ by inhibiting its metabolism and elimination and enhancing its oral absorption [52]. Intake of piperine can also enhance the oral bioavailability and systemic exposure of phenytoin (PNT), another common conventional antiepileptic drug, by inhibiting its hepatic metabolism and enhancing its oral absorption [53].

St John’s wort (SJW), the most commonly used herbal antidepressant, usually is taken at a dose of 900 mg/day, which is equivalent to approximately 40 mg/day of hyperforin. This high dose of hyperforin from SJW extracts is well-known as CYP3A4 and P-gp inducer [54]. However, the composition and yield of hyperforin and other ingredients, such as hypericin and quercetin, from SJW extracts are determined by the extraction methods [55]. For standardization of major compounds, hyperforin, SJW is commonly extracted by using hydroalcoholic solvents containing ethanol (>50%, *v*/*v*) in the commercial manufacturing process [56]. Therefore, the identification of extraction methods for SJW extracts or their composition is important to determine and predict possible NDIs exactly. Co-administration of SJW extracts (18.4 mg of hyperforin, 92.0 μg of hypericin, and 262 μg of pseudohypericin per 300 mg dried extract; Jarsin 300, Lichtwer Pharma AG, Berlin, Germany) can reduce the oral absorption and systemic exposure of amitriptyline due to induction of P-gp expression in the intestine and CYP3A4 expression in both intestine and liver of patients with depressive disease [57]. Moreover, a combination of SJW extracts (9–19 mg of hyperforin and 0.36–0.84 mg of hypericin per 300 mg dried extract; Hyperiplant, VSM Geneesmiddelen BV, Alkmaar, Netherlands) and docetaxel (DCT), one of the anti-cancer drugs for brain tumors, can cause significant decrease in the AUC value of DCT and significant increase of its systemic clearance by upregulating the expression of CYP3A4 in the liver and P-gp in the intestine, hepato-biliary membrane, and BBB of cancer patients by SJW extracts [58]. *Ginkgo biloba* (GB) extracts, which have antiplatelet, antioxidant, and neuroprotective activities, commonly contain 24% flavonol glycosides and 6% terpene lactones as major ingredients [59]. Flavonol glycosides in GB extracts are quercetin (QUE) or kaempferol conjugated with glucose or rhamnose. Terpene lactones include ginkgolides (3.1%) and bilobalide (2.9%), which are distinctive ingredients of GB extracts [60].GB Co-administration of GB extracts can enhance systemic exposure of midazolam by 25% and reduce its oral clearance by 26% due to the inhibitory effect on CYP3A4 in humans [61]. 

Garlic compounds are also widely intaken ingredients as a spice or immune-booster. However, intake of this garlic extract (up to 3.6 g/day via oral route) containing alliin, allicin, and *S*-allyl-l-cysteine may not significantly change the expression and activities of CYP2D6 and CYP3A4, leading to no relevant oral pharmacokinetic interaction with alprazolam [62].

In some countries, traditional formulations composed of various herb medicines have been frequently administered to the elderly or patients with cognitive and/or memorial impairment. Smart soup consisting of Rhizoma Acori Tatarinowii, Poria cum Radix Pini, and Radix Polygalae, a traditional Chinese medicine formula, can exhibit a synergistic effect with anti-AD agents. Co-administration of smart soup and donepezil, one of the cholinesterase inhibitors (ChEI), can reduce, by much more, the accumulation of amyloid plaque and prevent neurodegeneration in the brain of *drosophila* model compared to single donepezil treatment [63]. Moreover, this co-administration can more improve cognitive function in AD patients via pharmacodynamics NDIs, but the exact mechanism is still unknown [63]. In a similar way, co-administration of kihito extract, a traditional Japanese kampo medicine composed of *Ginseng Radix*, *Polygalae Radix*, *Astragali Radix*, *Zizyphi Fructus*, *Zizyphi Spinosi Semen*, *Angelicae Radix*, *Glycyrrhizae Radix*, *Atractylodis Rhizoma*, *Zingiberis Rhizoma*, Poria, *Saussureae Radix*, and *Longan Arillus* and ChEIs can also more improve cognitive function in AD patients compared to single ChEI treatment, but still exact mechanism should be addressed through further study [64].

**Table 1:** Clinical usage of natural compounds combined with various drugs for brain disorders.

Natural Compounds (Dose; Route)	Drug Molecules (Dose; Route)	Disease Models (Patients)	Refs.
Grapefruit juice (300 mL; Oral) Orange juice (200 mL; Oral)	CBZ (200 mg × 3; Oral)	Epileptic patients	[48,49]
Resveratrol (500 mg; Oral), Diosmin (500 mg; Oral)	CBZ (200 mg; Oral)	Healthy volunteers	[50,51]
Piperine (20 mg; Oral)	CBZ (500 mg; Oral), PNT (150 or 200 mg; Oral)	Epileptic patients	[52,53]
SJW extracts (300 mg × 3; Oral)	Amitriptyline (75 mg × 2; Oral)	Patients with depressant disease	[57]
SJW extracts (300 mg × 3; Oral)	DCT (135 mg; IV)	Cancer patients including brain tumor	[58]
GB extracts (120 mg × 3; Oral)	Midazolam (8 mg; Oral)	Healthy volunteers	[61]
Garlic extract (1.8 g × 2; Oral)	Alprazolam (2 mg; Oral)	Healthy volunteers	[62]
Smart soup; AT (15 g; Oral) + PRP (15 g; Oral) + RP (6 g; Oral)	Donepezil (5 mg; Oral)	Transgenic *drosophila* model and AD patients	[63]
Kihito extracts (2.5 g × 3; Oral)	ChEI (Oral)	AD patients	[64]

### 3.2. Pharmacokinetic and Pharmacodynamic NDIs in Brain Disorders

In preclinical investigations on NDIs, various pharmacokinetic NDIs, mainly associated with CYP enzymes in brain disorders, and pharmacodynamic NDIs have been studied extensively (Table 2). 

As mentioned previously, conventional antiepileptic drugs, such as CBZ and PNT, have been considered the likely candidates causing NDIs due to their extensive CYP3A4-mediated metabolism. Co-administration of *Polygonum cuspidatum*, a resveratrol-abundant dietary compound, enhanced the AUC value of CBZ 2–3 times and also enhanced its accumulation in the brain 4 times, which is similar to the interaction between single resveratrol and CBZ [50,65]. This NDI is mainly attributed to the inhibition of CYP3A4 in the liver and MRP2 in the renal tubule cells, although MRP2-mediated efflux in the brain is negligible [65]. *Paeonia emodi*, a well-known dietary compound for epileptic patients, can markedly enhance the Cmax, AUC and half-life of CBZ and reduce its oral clearance (CL/F; systemic clearance-to-oral bioavailability ratio) by downregulating the expression of hepatic CYP3A and CYP2C by approximately 50% when co-administered with CBZ [66]. In addition, the intake of sinapic acid, another well-known dietary compound for epileptic patients, can also markedly enhance the Cmax, AUC and half-life of CBZ and reduce its elimination constant and CL/F by inhibiting CYP3A2 and CYP2C11 in the liver and P-gp-mediated efflux transportation in the intestine [67]. In addition, co-administration of garden cress (*Lepidium sativum*) can increase both the Cmax and AUC of PNT by reducing its CL/F and prolonging its blood circulation [68].

However, some natural compounds can inhibit the antiepileptic effects of CBZ or PNT. Intake of GB extracts can reduce oral bioavailability and systemic exposure of CBZ and enhance its elimination rate in rats [69]. Ginsenosides are major composites of Ginseng formulations, which have been widely used in traditional herbal medicine; they exhibit anti-inflammatory and anti-tumor activities and improve cognitive function [70]. Among the ginsenosides, co-administration with protopanaxadiol (PPD), 25-OH-PPD, or 25-OCH_3_-PPD increased CYP3A4-mediated metabolism of CBZ in a human liver microsome study [71]. The intake of black seed (*Nigella sativa*) can drastically reduce the Cmax, AUC, and half-life of PNT by increasing its CL/F [68].

Imperatorin, a furocoumarin compound found in the roots of *Angelica sinensis* and *Angelica dahurica*, can enhance the Cmax and AUC of diazepam and reduce its CL/F by inhibiting its CYP-mediated hepatic metabolism, when co-administered with diazepam [72].

Khat (*Cathe edulis*), a well-known stimulant of the central nervous system (CNS), is consumed daily by approximately 80% of the adult population in Yemen, and this consumption has been expanded to other countries. Khat exerts potent inhibitory effects on CYP3A4, which is responsible for the metabolism of aripiprazole and vilazodone, and CYP2D6, which metabolizes aripiprazole and clomipramine [73]. Consequently, co-administration of Khat can enhance the Cmax and AUC of these drugs and reduce their systemic clearance and volume of distribution [74].

In a previous study, the pharmacokinetics of a drug exhibited a biphasic pattern depending on the dose of Ginseng. At a low dose of the *Panax ginseng* (PG) extract, the oral bioavailability and systemic exposure of selegiline were reduced by CYP1A2 induction, whereas at a high dose of the extract, the aforementioned parameters were enhanced due to the inhibition of CYP3A4 [75].

Furthermore, several natural compounds such as caffeine and QUE may cause pharmacodynamic NDIs with drugs for brain disorders. Caffeine, the most commonly consumed CNS-stimulant, was injected intraperitoneally (IP) at doses of 11.6, 23.1, and 46.2 mg/kg to mice with electroshock-induced convulsion. The intake of caffeine reduced the antiepileptic effects of CBZ, PNT, valproate, and topiramate in a dose-dependent manner without changes in the plasma concentrations of the drugs [76]. QUE, a flavonol found in many plants, is frequently consumed by patients with brain disorders owing to its various pharmacological effects, such as antioxidant, anti-inflammatory, and neuroprotective activities [77]. Co-administration of QUE with doxorubicin and temozolomide can exhibit synergistic anticancer effects pharmacodynamically in neuroblastoma and human astrocytoma cell lines, respectively, mainly due to the inhibitory effect of QUE on the expression of heat shock proteins [78,79]. Possible pharmacodynamics NDIs with synergistic effects in brain disorders are explored in detail in the following section (Section 3.4).

**Table 2:** Various natural compound–drug interactions (NDIs) and their effects on pharmacokinetic properties in brain disorders.

Natural Compounds (Dose; Route)	Drug Molecules (Dose; Route)	Observed Effects by Natural Compounds	Disease Models	Ref.
*Polygonum cuspidatum* (2 g/kg; Oral)	CBZ (200 mg/kg; Oral)	Enhancement of systemic exposure and brain concentration of CBZ, Inhibition of CYP3A-mediated metabolism and MRP2-mediated renal secretion.	Intact Sprague Dawley (SD) rats, MDCKII-MRP 2 cells	[65]
*Paeonia emodi* (200 mg/kg; Oral)	CBZ (80 mg/kg; Oral)	Enhancement of systemic exposure and prolongation of blood circulation of CBZ, Reduction in oral clearance, Downregulation of hepatic CYP3A2 and CYP2C11 expression.	Intact wistar rats	[66]
Sinapic acid (20 mg/kg; Oral)	CBZ (80 mg/kg; Oral)	Enhancement of systemic exposure and prolongation of blood circulation of CBZ, Reduction in drug elimination constant and oral clearance, Downregulation of hepatic CYP3A2, CYP2C11, and intestinal P-gp expression	Intact wistar rats	[67]
Garden cress (7.5 g; Oral)	PNT (50 mg, Oral)	Enhancement of systemic exposure and prolongation of blood circulation of PNT, Reduction in oral clearance.	Intact beagle dogs	[68]
GB extracts (Oral)	CBZ (Oral)	Reduction in oral bioavailability and systemic exposure of CBZ, Increase in elimination rate of CBZ.	Intact rats	[69]
Ginsenosides (1–50 μM)	CBZ (1 mM)	Increase in CBZ metabolism by inducing CYP3A4 activity	Human liver microsomes	[71]
Black seed (2.5 g; Oral)	PNT (50 mg, Oral)	Reduction in systemic exposure and blood circulation of PNT, Drastic increase in oral clearance.	Intact beagle dogs	[68]
Imperatorin (50 mg/kg; Oral)	Diazepam (10 mg/kg; Oral)	Enhancement of systemic exposure of diazepam, Reduction in oral clearance, Inhibition of hepatic metabolism.	Intact SD rats	[72]
Khat extracts (300 mg/kg; IP)	Clomipramine (10 mg/kg; IP), Vilazodone (10 mg/kg; IP), Aripiprazole (10 mg/kg; IP)	Enhancement of systemic exposure of these drugs, Reduction in oral clearance and volume of distribution.	Intact SD rats	[74]
PG extracts (1 g/kg, 3 g/kg; Oral)	Selegiline (30 mg/kg; Oral)	At a low dose of PG extract, induction of CYP1A2 caused reduction in oral bioavailability and systemic exposure of selegiline. At a high dose of PG extract, oral bioavailability and systemic exposure of selegiline were enhanced due to the inhibition of CYP3A4.	Intact SD rats	[75]

### 3.3. NDIs Affecting Drug Transporters in the Brain

**Figure 3:**
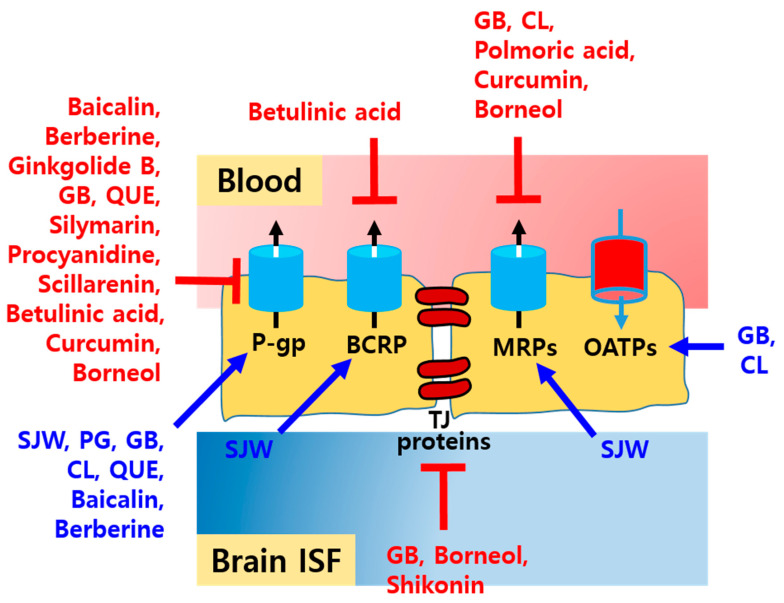
Modulation of drug transporters by natural compounds in the brain.

Among pharmacokinetic NDIs, the interactions associated with drug transporters, such as ABC and SLC transporters, have also been widely investigated, although P-gp in the BBB has been the focus. The modulation of ABC and SLC transporters by natural compounds in the brain is summarized in Table 3 and Figure 3. 

Continuous intake of SJW extracts with a high dose of hyperforin ([80]; 5% hyperforin per 300 mg of dried extract, Dr. Wilmar Schwabe Pharmaceuticals, Karlsruhe, Germany and [81]; 0.61% hyperforin and 0.3% hypericin per 300 mg of dried extract, Finzelberg GmbH and Co. KG, Andernach, Germany) can upregulate P-gp, BCRP, and MRP2 by activating pregnane-X-receptor (PXR) in the rat hippocampus and the BBB in transgenic mice with AD [54,80,81]. In contrast to the effects observed as a result of the high dose of hyperforin, no significant pharmacokinetic interactions have been reported in clinical and experimental studies at a low dose of hyperforin (≤1 mg/day) [82]. 

Intake of PG can also upregulate the expression of P-gp in the intestine and BBB of rats, thereby reducing systemic exposure to fexofenadine and its brain uptake [83]. In contrast, a previous study reported that the intake of Korean red ginseng extract at a dose of 0.5–3 g per day did not cause significant NDIs due to no relevant changes in the expression of drug-metabolizing enzymes and transporters in the brain [84]. Thus, further studies concerning the influence of PG on CYP enzymes in the liver and drug transporters in the intestine and brain are needed, taking into account its dose, composition, and duration of administration [81].

On the other hand, administration of ginkgolide B, a major component of terpenes in GB extracts, or ginkgolide B derivative (ginkgolide B pyrazine) can reduce the expression of P-gp in rat brain microvascular endothelial cells (rBMECs) and in the cerebral cortex of rats, thereby enhancing the brain uptake of rhodamine 123 (Rho 123) [85]. In addition, co-administration of GB extract could downregulate the expression of P-gp in the brain of P-gp-overexpressing mice with epilepsy, thereby enhancing the brain delivery of PNT [86]. Moreover, the intake of GB extract at a dose of 400 mg/kg can result in reversible BBB opening, thereby enhancing the brain uptake of ginsenosides administered orally at a dose of 500 mg/kg in mouse and rat models, without significant changes in TJ proteins and their serum concentration [87]. However, in a previous study, GB extract was reported to upregulate the expression of P-gp and downregulate MRP2 expression in the rat hippocampus [81]. Thus, further thorough studies concerning the influence of GB on the expression of drug transporters in the brain are also needed.

Similar to GB extract, intake of rhizome extract of *Curcuma longa* (CL) can upregulate the expression of P-gp, OATP1a5, and OATP1c1 and downregulate the expression of MRP1 and MRP2 in the rat hippocampus [88].

NDIs associated with drug transporters in the brain may exhibit a biphasic pattern depending on the dose of the natural compound. Studies have reported that baicalin and berberine can affect P-gp efflux of nimodipine in a bidirectional manner, which indicates that P-gp efflux of nimodipine can be inhibited at low doses of these natural compounds, and at a high dose, the uptake of nimodipine can be reduced in rBMECs [89]. It has also been reported that QUE can cause dose-dependent biphasic pharmacokinetic NDIs [90]. The uptake of vincristine into mouse brain capillary endothelial cells (mBECs) and its brain uptake in mice could be reduced at a low dose of QUE (5–10 μM in cells and 0.1 mg/kg in mice) due to the activation of P-gp in the BBB, whereas its uptake in both the cells and brain of mice could be enhanced at a high dose of QUE (25–50 μM in cells and 1.0 mg/kg in mice) by inhibiting P-gp in the BBB [91]. In accordance with previous studies, the intake of a high dose of QUE may affect P-gp-mediated efflux transportation in one direction. Co-administration of QUE can enhance the brain uptake of ritonavir and quinidine by inhibiting P-gp in the BBB [92,93].

Similar to QUE, co-administration of silymarin can enhance the systemic exposure and brain uptake of quinidine by inhibiting P-gp in both the intestine and BBB [93].

Some natural compounds can be administered with anticancer agents for brain tumors, and this co-administration can affect the pharmacokinetics of drugs by altering the expression of efflux transporters in the brain. Co-administration of procyanidin, which is isolated from the bark of *Pinus massoniana*, can enhance the intracellular uptake of Rho 123 into rBMECs by inhibiting P-gp in the BBB [94]. In addition, based on the same mechanism, procyanidin can exert synergistic anticancer effects with doxorubicin (DOX) due to the enhanced brain delivery of DOX in human cerebroma cell-transplanted mice [94]. Furthermore, scillarenin, polmoric acid, and betulinic acid, which possess anticancer activity, can also downregulate the expression of P-gp, MRP1, P-gp and BCRP, respectively, in both brain cancer cells and the BBB [95,96,97].

**Table 3:** Various NDIs and their effects on drug transporters in the brain.

Natural Compounds (Dose; Route)	Drug Molecules (Dose; Route)	Observed Effects by Natural Compounds	Disease Models	Refs.
SJW extracts (300 mg/kg, 1250 mg/kg; Oral)	-	Induction of P-gp, BCRP, and MRP2 by activating PXR.	Intact wistar rats, Transgenic mice with AD	[54,80,81]
PG suspension (150 mg/kg; Oral)	Fexofenadine (100 mg/kg; Oral)	Reduction in oral bioavailability, systemic exposure, and brain uptake of fexofenadine, Induction of P-gp expression in the intestine and the BBB.	Intact SD rats	[83]
Ginkgolide B, Ginkgolide B derivative (10 mg/kg; IV), (5–100 μM)	Rho 123 (0.2 mg/kg; IV) -	Downregulation of P-gp expression in cell levels and the rat cerebral cortex, thereby enhancing delivery of the molecules to the brain.	Intact SD rats and rBMECs	[85]
GB extracts (30 mg/kg; IP)	PNT (40 mg/kg; Oral)	Downregulation of P-gp expression in the brain of mice, thereby enhancing delivery of PNT to the brain.	P-gp- overexpressed mice with epilepsy	[86]
GB extracts (200 mg/kg; Oral)	-	Upregulation of P-gp, OATP1a4, and OATP1a5 expression and downregulation of MRP2 expression in the rat hippocampus.	Intact wistar rats	[81]
Rhizome extract of CL (500 mg/kg; Oral)	-	Upregulation of P-gp, OATP1a5, and OATP1c1 expression and downregulation of MRP1 and MRP2 expression in the rat hippocampus	Intact wistar rats	[88]
QUE (0.1 mg/kg, 1.0 mg/kg; IV), (5–50 μM)	Vincristine (1.0 mg/kg; IV), (30 nM)	At a low dose of QUE, the uptake of vincristine into mBECs and its brain-to-plasma concentration ratio in the mice were reduced due to activation of P-gp in the BBB, At a high dose of QUE, the uptake of vincristine into mBECs and its brain-to-plasma concentration ratio in the mice were enhanced by inhibiting P-gp in the BBB.	ddY mice, mBECs	[91]
QUE (100 mg/kg; Oral), (25 μM)	Ritonavir (20 mg/kg; Oral), (50 μM)	Enhancement of ritonavir uptake into human BMECs and its brain-to-plasma concentration ratio in the rats by inhibiting P-gp in the BBB.	Intact SD rats, Human BMECs	[92]
QUE (20 mg/kg; IV), Silymarin (20 mg/kg; IV)	Quinidine (5 mg/kg; IV)	Enhancement of systemic exposure, brain concentration, and brain-to-plasma concentration ratio of quinidine, Inhibition of P-gp-mediated efflux transport in the BBB.	Intact C57 mice	[93]
Procyanidine (0.5–10 μM), (80 mg/kg; IP)	Rho 123 (10 μM), DOX (2 mg/kg; IV)	Enhancement of Rho 123 uptake into rBMECs by inhibiting P-gp in the BBB, Synergistic anticancer efficacy of DOX due to the enhanced delivery of DOX to the brain by inhibiting P-gp.	rBMECs, Human cerebroma cell-transplanted mice	[94]

### 3.4. Combinatorial Therapies of Natural Compound and Drug for Synergistic Effects in Brain Disorders

Combinatorial therapies of natural compounds and drugs have been applied for synergistic effects in brain disorders using pharmacokinetic/pharmacodynamic NDIs. Natural compounds causing potentiate pharmacokinetic drug interactions associated with the inhibition of CYP metabolism, renal excretion, and/or efflux transportation in the intestine and brain and causing synergistic pharmacodynamic drug interactions can be co-administered or formulated with drugs for the enhanced treatment of brain disorders. 

As aforementioned, *Polygonum cuspidatum*, *Paeonia emodi*, Garden cress, Khat, resveratrol, diosmin, piperine, sinapic acid, imperatorin, and grapefruit juice can inhibit CYP-mediated metabolism of drugs for brain disorders, thereby enhancing their systemic exposure and/or brain-to-plasma concentration ratio, leading to raising their pharmacological effects against brain disorders. In addition, natural compounds, such as QUE, can cause pharmacodynamic NDIs with drugs for brain disorders, thereby exerting synergistic therapeutic effects as mentioned earlier. Moreover, co-administration of melatonin, an endogenous molecule for circadian cycle and homeostasis, exerted synergistic antiepileptic effects with CBZ, PNT, and phenobarbital in mice with electroshock-induced convulsion and neonatal rats without changes in their plasma concentration [98,99]. Curcumin found in CL extract, which possesses various pharmacological activities including antioxidant, anti-inflammatory, anti-tumor, antidepressant, liver-protective, and neuroprotective effects, also enhanced antiepileptic effects of CBZ, PNT, valproate, and phenobarbital with improvement on cognitive function and neuroprotection, whereas it did not significantly change the plasma concentration of the drugs [100]. Co-administration of docosahexaenoic acid, an abundant endogenous biomolecule in the brain, can exert synergistic antiepileptic effects with both valproate and lamotrigine in pentylenetetrazole and kindling models of epilepsy [101].

Natural compounds that have been demonstrated as a potent inhibitor of P-gp, BCRP, and MRP transporters on the BBB can improve the therapeutic effects of drugs for brain disorders, particularly in the case of brain cancer, by enhancing BBB permeability, leading to the accumulation of the drugs in the brain [102]. As aforementioned, co-administration of procyanidin, scillarenin, polmoric acid, betulinic acid, and a high dose of QUE could enhance the efficacy of anticancer agents against brain cancer by inhibiting efflux transporters in the BBB. A recent study reported that curcumin nanoparticles loaded with DOX could more efficiently deliver and localize DOX into P-gp- and MRP-overexpressed cancer cells (e.g., glioblastoma) by inhibiting those efflux transporters, thereby enhancing the anticancer efficacy in the tumor-xenograft model compared to the treatment of DOX solution, while this synergistic effect is the most remarkably exhibited in the treatment of polyethylene glycol-conjugated curcumin nanoparticles loaded with DOX [103]. 

Furthermore, efflux transporter-inhibiting natural compounds can be used as potentiators of drugs for other brain disorders, such as ischemic stroke and neurodegenerative diseases. Co-administration of shikonin, a major composite from *Lithospermum erythrorhizon*, can inhibit the expression of matrix metalloproteinase-9 in the damaged brain and enhance BBB permeability of anti-stroke drugs in focal cerebral ischemia-induced mice [104]. A recent study reported that betulinic acid nanoparticles loaded with glyburide could enhance the brain delivery of glyburide by inhibiting P-gp and BCRP in the BBB, thereby exerting a higher therapeutic effect in the middle cerebral artery occlusion model, compared to the treatment of glyburide solution [105]. Borneol, a bicyclic monoterpene that exerts anti-inflammatory and antiepileptic activities, possesses BBB permeability-enhancing effect by inhibiting P-gp and MRPs in the BBB and disassembling TJ proteins, reversibly [106]. Recent studies reported that borneol-modified nanoparticles and niosomes could enhance the brain uptake of loaded drugs, dopamine, and ginkgolide B and puerarin, respectively, compared to the plane nanoparticle and plane niosomes, thereby exerting more potent therapeutic effects in Parkinson’s disease-induced model [107,108].

## 4. Challenges of NDIs and Future Remarks 

Although the global market size of natural compounds and phytochemicals for alternative and preservative medicines has been up to USD 83 billion in 2008, and the consumer worldwide has increased in exponential progression owing to their well-known safety and somewhat apparent efficacy, estimation and prediction of NDIs have been relatively more restricted compared to in case of drug–drug interactions due to the scant pharmacokinetic studies, dose-dependent studies, randomized clinical trials, and regulatory process including constituents identification, ingredient standardization, and quality control for natural compounds [4,109]. In addition, the lack of knowledge on the localization of various transporters in the BBB and BCSFB of the human brain and uncertain information regarding substrates (e.g., natural compounds)–transporters complex can more hinder the exact determination of NDIs [110]. While many preclinical and clinical studies have demonstrated the potential for CYP- and/or P-gp-mediated NDIs, NDIs related with other drug transporters excepting P-gp, phase II metabolism, and transporters-mediated renal excretion are still missing [46]. Moreover, the discrepancy between in vitro estimation and in vivo studies for NDIs (i.e., lack of in vitro–in vivo correlation), and difference with animal models and humans regarding the expression of drug transporters and the consequent NDIs can also impede the exact prediction of NDIs in clinical [111]. Moreover, considering that, on occasion, drug concentration in blood, plasma, or CSF does not represent the concentration into the brain tissues or ISF, NDIs in the BBB and BCSFB may not significantly change its plasma concentration, whereas NDIs can affect the drug concentration in the brain and its efficacy against brain disorders [4,112]. Therefore, for the exact determination and prediction of NDIs, other kinds of measuring methods like brain imaging by using positron emission tomography and magnetic resonance spectroscopy are considered as a more adequate and relevant tool instead of drug quantification through blood or CSF collection, in recent times [16,113]. Moreover, several recent studies reported that deep learning models, kinds of in silico method for prediction of NDIs, can exhibit improved accuracy and more efficient performance, insisting that these models may play a key role in future research, drug discovery, and development processes to estimate possible interactions between natural compounds and new drugs for brain disorders [114,115,116]. 

## Data Availability

Data is contained within the article.

## Abbreviations

NDI	Natural compound–drug interaction
BBB	Blood–brain barrier
BCSFB	Blood–cerebrospinal fluid barrier
ADME	Absorption, distribution, metabolism, and excretion
ISF	Interstitial fluid
CSF	Cerebrospinal fluid
CP	Choroid plexus
TJ	Tight junction
AJ	Adherence junction
JAM	Junctional adhesion molecule
ZO	Zonula occluden
CYP	Cytochrome P450
SLC	Solute carrier
ATP	Adenosine triphosphate
OATP	Organic anion transporting polypeptide
OAT	Organic anion transporter
OCT	Organic cation transporter
LAT	l-amino acid transporter
PEPT	Peptide transporter
GLUT	Glucose transporter
OCTN	Novel organic cation transporter
ABC	ATP-binding cassette
P-gp	P-glycoprotein
MRP	Multidrug resistance-associated protein
BCRP	Breast cancer resistance protein
GI	Gastrointestinal
BSEP	Bile salt export pump
MATE	Multidrug and toxin extrusion transporter
AD	Alzheimer’s disease
CBZ	Carbamazepine
PNT	Phenytoin
SJW	St John’s wort
DCT	Docetaxel
GB	Ginkgo biloba
ChEI	Cholinesterase inhibitor
CL	Systemic clearance
F	Oral bioavailability
PPD	Protopanaxadiol
CNS	Central nervous system
PG	Panax ginseng
QUE	Quercetin
PXR	Pregnane-X-receptor
BMEC	Brain microvascular endothelial cell
Rho	Rhodamine
CL	Curcuma longa
BEC	Brain capillary endothelial cell
DOX	Doxorubicin

## References

[1] Kim K.T., Lee H.S., Lee J.J., Park E.K., Lee B.S., Lee J.Y., Bae J.S. (2018). Nanodelivery systems for overcoming limited transportation of therapeutic molecules through the blood-brain barrier. Future Med. Chem..

[2] Makkar R., Behl T., Bungau S., Zengin G., Mehta V., Kumar A., Uddin M.S., Ashraf G.M., Abdel-Daim M.M., Arora S. (2020). Nutraceuticals in neurological disorders. Int. J. Mol. Sci..

[3] Uddin M.S., Hossain M.F., Mamun A.A., Shah M.A., Hasana S., Bulbul I.J., Sarwar M.S., Mansouri R.A., Ashraf G.M., Rauf A. (2020). Exploring the multimodal role of phytochemicals in the modulation of cellular signaling pathways to combat age-related neurodegeneration. Sci. Total Environ..

[4] Kibathi L.W., Bae S.H., Penzak S.R., Kumar P. (2018). Potential influence of centrally acting herbal drugs on transporters at the blood-cerebrospinal fluid barrier and blood-brain barrier. Eur. J. Drug Metab. Pharmacokinet..

[5] Khadka B., Lee J.Y., Park D.H., Kim K.T., Bae J.S. (2020). The role of natural compounds and their nanocarriers in the treatment of CNS inflammation. Biomolecules.

[6] Borse S.P., Singh D.P., Nivsarkar M. (2019). Understanding the relevance of herb-drug interaction studies with special focus on interplays: A prerequisite for integrative medicine. Porto Biomed. J..

[7] Roberts A.G., Gibbs M.E. (2018). Mechanisms and the clinician relevance of complex drug-drug interactions. Clin. Pharmacol..

[8] Qosa H., Miller D.S., Pasinelli P., Trotti D. (2015). Regulation of ABC efflux transporters at blood-brain barrier in health and neurological disorders. Brain Res..

[9] Vengoji R., Macha M.A., Batra S.K., Shonka N.A. (2018). Natural products: A hope for glioblastoma patients. Oncotarget.

[10] König J., Müller F., Fromm M.F. (2013). Transporters and drug-drug interactions: Important determinants of drug disposition and effects. Pharmacol. Rev..

[11] Redzic Z. (2011). Molecular biology of the blood-brain and the blood-cerebrospinal fluid barriers: Similarities and differences. Fluids Barriers CNS.

[12] Tietz S., Engelhardt B. (2015). Brain barriers: Crosstalk between complex tight junctions and adherens junctions. J. Cell Biol..

[13] Dias M.C., Mapunda J.A., Vladymyrov M., Engelhardt B. (2019). Structure and junctional complexes of endothelial, epithelial and glial brain barriers. Int. J. Mol. Sci..

[14] Parashar P., Diwaker N., Kanoujia J., Singh M., Yadav A., Singh I., Saraf S.A. (2020). In situ gel of lamotrigine for augmented brain delivery: Development, characterization and pharmacokinetic evaluation. J. Pharm. Investig..

[15] Agúndez J.A., Jiménez-Jiménez F.J., Alonso-Navarro H., García-Martín E. (2014). Drug and xenobiotic biotransformation in the blood-brain barrier: A neglected issue. Front. Cell. Neurosci..

[16] Eyal S., Hsiao P., Unadkat J.D. (2009). Drug interactions at the blood-brain barrier: Fact or fantasy?. Pharmacol. Ther..

[17] Chan G.N., Hoque T., Bendayan R. (2013). Role of nuclear receptors in the regulation of drug transporters in the brain. Trends Pharmacol. Sci..

[18] Han D.G., Cho S.S., Kwak J.H., Yoon I.S. (2019). Medicinal plants and phytochemicals for diabetes mellitus: Pharmacokinetic characteristics and herb-drug interactions. J. Pharm. Investig..

[19] Müller F., Fromm M.F. (2011). Transporter-mediated drug-drug interactions. Pharmacogenomics.

[20] Giacomini K.M., Huang S.M., Tweedie D.J., Benet L.Z., Brouwer K.L., Chu X., Dahlin A., Evers R., Fischer V., International Transporter Consortium (2010). Membrane transporters in drug development. Nat. Rev. Drug Discov..

[21] Jeong S.H., Jang J.H., Cho H.Y., Oh I.J., Lee Y.B. (2020). A sensitive UPLC-ESI-MS/MS method for the quantification of cinnamic acid in vivo and in vitro: Application to pharmacokinetic and protein binding study in human plasma. J. Pharm. Investig..

[22] Singh A., Zhao K. (2017). Herb-drug interactions of commonly used Chinese medicinal herbs. Int. Rev. Neurobiol..

[23] Sewradj S., Braver M., Vermeulen N., Commandeur J., Richert L., Vos J. (2016). Inter-donor variability of phase I/phase II metabolism of three reference drugs in cryopreserved primary human hepatocytes in suspension and monolayer. Toxicol. In Vitro.

[24] Pandit S., Kanjilal S., Awasthi A., Chaudhary A., Banerjee D., Bhatt B.N., Narwaria A., Singh R., Dutta K., Jaggi M. (2017). Evaluation of herb-drug interaction of a polyherbal Ayurvedic formulation through high throughput cytochrome P450 enzyme inhibition assay. J. Ethnopharmacol..

[25] Thelen K., Dressman J.B. (2009). Cytochrome P450-mediated metabolism in the human gut wall. J. Pharm. Pharmacol..

[26] Xie F., Ding X., Zhang Q.Y. (2016). An update on the role of intestinal cytochrome P450 enzymes in drug disposition. Acta Pharm. Sin. B.

[27] Ghosh C., Puvenna V., Gonzalez-Martinez J., Janigro D., Marchi N. (2011). Blood-brain barrier P450 enzymes and multidrug transporters in drug resistance: A synergistic role in neurological diseases. Curr. Drug Metab..

[28] Naumann S., Haller D., Eisner P., Schweiggert-Weisz U. (2020). Mechanisms of interactions between bile acids and plant compounds-A review. Int. J. Mol. Sci..

[29] Wu X., Ma J., Ye Y., Lin G. (2016). Transporter modulation by Chinese herbal medicines and its mediated pharmacokinetic herb-drug interactions. J. Chromatogr. B.

[30] Yin J., Wang J. (2016). Renal drug transporters and their significance in drug-drug interactions. Acta Pharm. Sin. B.

[31] Ivanyuk A., Livio F., Biollaz J., Buclin T. (2017). Renal drug transporters and drug interactions. Clin. Pharmacokinet..

[32] Niu J., Straubinger R.M., Mager D.E. (2019). Pharmacodynamic drug-drug interactions. Clin. Pharmacol. Ther..

[33] Efferth T., Koch E. (2011). Complex interactions between phytochemicals. The multi-target therapeutic concept of phytotherapy. Curr. Drug Targets.

[34] Caesar L.K., Cech N.B. (2019). Synergy and antagonism in natural product extracts: When 1+1 dose not equal 2. Nat. Prod. Rep..

[35] Vakil V., Trappe W. (2019). Drug combinations: Mathematical modeling and networking methods. Pharmaceutics.

[36] Erickson M.A., Banks W.A. (2019). Age-associated changes in the immune system and blood-brain barrier functions. Int. J. Mol. Sci..

[37] Chen R.L., Kassem N.A., Redzic Z.B., Chen C.P., Segal M.B., Preston J.E. (2009). Age-related changes in choroid plexus and blood-cerebrospinal fluid barrier function in the sheep. Exp. Gerontol..

[38] Obermeier B., Daneman R., Ransohoff R.M. (2013). Development, maintenance and disruption of the blood-brain barrier. Nat. Med..

[39] Gomez-Zepeda D., Taghi M., Scherrmann J.M., Decleves X., Menet M.C. (2020). ABC transporters at the blood-brain interfaces, their study models, and drug delivery implications in gliomas. Pharmaceutics.

[40] Wang G.X., Wang D.W., Liu Y., Ma Y.H. (2016). Intractable epilepsy and the P-glycoprotein hypothesis. Int. J. Neurosci..

[41] DeMars K.M., Yang C., Hawkins K.E., McCrea A.O., Siwarski D.M., Candelario-Jalil E. (2017). Spatiotemporal changes in p-glycoprotein levels in brain and peripheral tissues following ischemic stroke in rats. J. Exp. Neurosci..

[42] Erdö F., Krajcsi P. (2019). Age-related functional and expressional changes in efflux pathways at the blood-brain barrier. Front. Aging Neurosci..

[43] Qosa H., Mohamed L.A., Alqahtani S., Abuasal B.S., Hill R.A., Kaddoumi A. (2016). Transporters as drug targets in neurological diseases. Clin. Pharmacol. Ther..

[44] Matsuo H., Tomiyama H., Satake W., Chiba T., Onoue H., Kawamura Y., Nakayama A., Shimizu S., Sakiyama M., Funayama M. (2015). ABCG2 variant has opposing effects on onset ages of Parkinson’s disease and gout. Ann. Clin. Transl. Neurol..

[45] Ohtsuki S., Yamaguchi H., Kang Y.S., Hori S., Terasaki T. (2010). Reduction of L-type amino acid transporter 1 mRNA expression in brain capillaries in a mouse model of Parkinson’s disease. Biol. Pharm. Bull..

[46] Rombolà L., Scuteri D., Marilisa S., Watanabe C., Morrone L.A., Bagetta G., Corasaniti M.T. (2020). Pharmacokinetic interactions between herbal medicines and drugs: Their mechanisms and clinical relevance. Life.

[47] Fong S.Y., Gao Q., Zuo Z. (2013). Interaction of carbamazepine with herbs, dietary supplements, and food: A systematic review. Evid. Based Complement. Altern. Med..

[48] Garg S.K., Kumar N., Bhargava V.K., Prabhakar S.K. (1998). Effect of grapefruit juice on carbamazepine bioavailability in patients with epilepsy. Clin. Pharmacol. Ther..

[49] Bhagat A., Bavaskar S., Tamboli J.A., Nighute A.B., Bhise S.B. (2009). Effect of orange juice on the bioavailability of carbamazepine. J. Pharm. Res..

[50] Bedada S.K., Nearati P. (2015). Effect of resveratrol on the pharmacokinetics of carbamazepine in healthy human volunteers. Phytother. Res..

[51] Bedada S.K., Boga P.K. (2017). Influence of diosmin on the metabolism and disposition of carbamazepine in healthy subjects. Xenobiotica.

[52] Pattanaik S., Hota D., Prabhakar S., Kharbanda P., Pandhi P. (2009). Pharmacokinetic interaction of single dose of piperine with steady-state carbamazepine in epilepsy patients. Phytother. Res..

[53] Pattanaik S., Hota D., Prabhakar S., Kharbanda P., Pandhi P. (2006). Effect of piperine on the steady-state pharmacokinetics of phenytoin in patients with epilepsy. Phytother. Res..

[54] Nicolussi S., Drewe J., Butterweck V., Meyer Zu Schwabedissen H.E. (2020). Clinical relevance of St. John’s wort drug interactions revisited. Br. J. Pharmacol..

[55] Gaid M., Biedermann E., Füller J., Haas P., Behrends S., Krull R., Scholl S., Wittstock U., Müller-Goymann C., Beerhues L. (2018). Biotechnological production of hyperforin for pharmaceutical formulation. Eur. J. Pharm. Biopharm..

[56] Novelli M., Masiello P., Beffy P., Menegazzi M. (2020). Protective role of St. John’s wort and its components hyperforin and hypericin against diabetes through inhibition of inflammatory signaling: Evidence from in vitro and in vivo studies. Int. J. Mol. Sci..

[57] Johne A., Schmider J., Brockmöller J., Stadelmann A.M., Störmer E., Bauer S., Scholler G., Langheinrich M., Roots I. (2002). Decreased plasma levels of amitriptyline and its metabolites on comedication with an extract from St. John’s wort (*Hypericum perforatum*). J. Clin. Psychopharmacol..

[58] Goey A.K., Meijerman I., Rosing H., Marchetti S., Mergui-Roelvink M., Keessen M., Burgers J.A., Beijnen J.H., Schellens J.H. (2014). The effect of St John’s wort on the pharmacokinetics of docetaxel. Clin. Pharmacokinet..

[59] Ražná K., Sawinska Z., Ivanišová E., Vukovic N., Terentjeva M., Stričík M., Kowalczewski P.Ł., Hlavačková L., Rovná K., Žiarovská J. (2020). Properties of *Ginkgo biloba* L.: Antioxidant characterization, antimicrobial activities, and genomic microRNA based marker fingerprints. Int. J. Mol. Sci..

[60] Yoshitake T., Yoshitake S., Kehr J. (2010). The *Ginkgo biloba* extract EGb 761^®^ and its main constituent flavonoids and ginkgolides increase extracellular dopamine levels in the rat prefrontal cortex. Br. J. Pharmacol..

[61] Uchida S., Yamada H., Li X.D., Maruyama S., Ohmori Y., Oki T., Watanabe H., Umegaki K., Ohashi K., Yamada S. (2006). Effects of Ginkgo biloba extract on pharmacokinetics and pharmacodynamics of tolbutamide and midazolam in healthy volunteers. J. Clin. Pharmacol..

[62] Markowitz J.S., Devane C.L., Chavin K.D., Taylor R.M., Ruan Y., Donovan J.L. (2003). Effects of garlic (*Allium sativum* L.) supplementation on cytochrome P450 2D6 and 3A4 activity in healthy volunteers. Clin. Pharmacol. Ther..

[63] Wang Y., Wang Y., Sui Y., Yu H., Shen X., Chen S., Pei G., Zhao J., Ding J. (2015). The combination of aricept with a traditional Chinese medicine formula, smart soup, may be a novel way to treat Alzheimer’s disease. J. Alzheimers Dis..

[64] Watari H., Shimada Y., Matsui M., Tohda C. (2019). Kihito, a traditional Japanese kampo medicine, improves cognitive function in Alzheimer’s disease patients. Evid. Based Complement. Altern. Med..

[65] Chi Y.C., Lin S.P., Hou Y.C. (2012). A new herb-drug interaction of *Polygonum cuspidatum*, a resveratrol-rich nutraceutical, with carbamazepine in rats. Toxicol. Appl. Pharmacol..

[66] Raish M., Ahmad A., Alkharfy K.M., Jan B.L., Mohsin K., Ahad A., Al-Jenoobi F.I., Al-Mohizea A.M. (2017). Effects of Paeonia emodi on hepatic cytochrome P450 (CYP3A2 and CYP2C11) expression and pharmacokinetics of carbamazepine in rats. Biomed. Pharmacother..

[67] Raish M., Ahmad A., Ansari M.A., Alkharfy K.M., Ahad A., Al-Jenoobi F.I., Al-Mohizea A.M., Khan A., Ali N. (2019). Effects of sinapic acid on hepatic cytochrome P450 3A2, 2C11, and intestinal P-glycoprotein on the pharmacokinetics of oral carbamazepine in rats: Potential food/herb-drug interaction. Epilepsy Res..

[68] Alkharfy K.M., Al-Jenoobi F.I., Al-Mohizea A.M., Al-Suwayeh S.A., Khan R.M., Ahmad A. (2013). Effects of Lepidium sativum, Nigella sativa and Trigonella foenum-graceum on phenytoin pharmacokinetics in beagle dogs. Phytother. Res..

[69] Chandra R.H., Rajkumar M., Veeresham C. (2009). Pharmacokinetic interaction of *Ginkgo biloba* with carbamazepine. Planta Med..

[70] Kim H.J., Kim P., Shin C.Y. (2013). A comprehensive review of the therapeutic and pharmacological effects of ginseng and ginsenosides in central nervous system. J. Ginseng Res..

[71] Hao M., Zhao Y., Chen P., Huang H., Liu H., Jiang H., Zhang R., Wang H. (2008). Structure-activity relationship and substrate-dependent phenomena in effects of ginsenosides on activities of drug-metabolizing P450 enzymes. PLoS ONE.

[72] Zhou Y., Meng D., Chen F., Wu Z., Wang B., Wang S., Geng P., Dai D., Zhou Q., Qiu W. (2020). Inhibitory effect of imperatorin on the pharmacokinetics of diazepam in vitro and in vivo. Front. Pharmacol..

[73] Bedada W., de Andrés F., Engidawork E., Pohanka A., Beck O., Bertilsson L., Llerena A., Aklillu E. (2015). The psychostimulant Khat (*Catha edulis*) inhibits CYP2D6 enzyme activity in humans. J. Clin. Psychopharmacol..

[74] Elkady E.F., Fouad M.A., Alshoba N., Mahmoud S.T. (2020). Validated LC-MS/MS method for the determination of some prescribed CNS drugs: Application to an *in vivo* pharmacokinetic study of drug-herb metabolic interaction potential of khat. Microchem. J..

[75] Yang L., Li C.L., Tsai T.H. (2020). Preclinical herb-drug pharmacokinetic interaction of *Panax ginseng* extract and selegiline in freely moving rats. ACS Omega.

[76] Chrościńska-Krawczyk M., Jargiełło-Baszak M., Wałek M., Tylus B., Czuczwar S.J. (2011). Caffeine and the anticonvulsant potency of antiepileptic drugs: Experimental and clinical data. Pharmacol. Rep..

[77] Pezzani R., Salehi B., Vitalini S., Iriti M., Zuñiga F.A., Sharifi-Rad J., Martorell M., Martins N. (2019). Synergistic effects of plant derivatives and conventional chemotherapeutic agents: An update on the cancer perspective. Medicina.

[78] Zanini C., Giribaldi G., Mandili G., Carta F., Crescenzio N., Bisaro B., Doria A., Foglia L., di Montezemolo L.C., Timeus F. (2007). Inhibition of heat shock proteins (HSP) expression by quercetin and differential doxorubicin sensitization in neuroblastoma and Ewing’s sarcoma cell lines. J. Neurochem..

[79] Jakubowicz-Gil J., Langner E., Wertel I., Piersiak T., Rzeski W. (2010). Temozolomide, quercetin and cell death in the MOGGCCM astrocytoma cell line. Chem. Biol. Interact..

[80] Brenn A., Grube M., Jedlitschky G., Fischer A., Strohmeier B., Eiden M., Keller M., Groschup M.H., Vogelgesang S.S. (2014). John’s Wort reduces beta-amyloid accumulation in a double transgenic Alzheimer’s disease mouse model-role of P-glycoprotein. Brain Pathol..

[81] Mrozikiewicz P.M., Bogacz A., Bartkowiak-Wieczorek J., Kujawski R., Mikolajczak P.L., Ozarowski M., Czerny B., Mrozikiewicz-Rakowska B., Grzeskowiak E. (2014). Screening for impact of popular herbs improving mental abilities on the transcriptional level of brain transporters. Acta Pharm..

[82] Zahner C., Kruttschnitt E., Uricher J., Lissy M., Hirsch M., Nicolussi S., Krähenbühl S., Drewe J. (2019). No clinically relevant interactions of St. John’s wort extract Ze 117 low in hyperforin with cytochrome P450 enzymes and P-glycoprotein. Clin. Pharmacol. Ther..

[83] Zhang R., Jie J., Zhou Y., Cao Z., Li W. (2009). Long-term effects of *Panax ginseng* on disposition of fexofenadine in rats in vivo. Am. J. Chin. Med..

[84] Choi M.K., Song I.S. (2019). Interactions of ginseng with therapeutic drugs. Arch. Pharm. Res..

[85] Hui A., Zhu S., Yin H., Yang L., Zhang Z., Zhou A., Pan J., Zhang W. (2016). Novel ginkgolide B derivative attenuated the function and expression of P-glycoprotein at the blood-brain barrier, presenting brain-targeting ability. RSC Adv..

[86] Zhang C., Fan Q., Chen S.L., Ma H. (2015). Reversal of P-glycoprotein overexpression by *Ginkgo biloba* extract in the brain of pentylenetetrazole-kindled and phenytoin-treated mice. Kaohsiung J. Med. Sci..

[87] Liang W., Xu W., Zhu J., Zhu Y., Gu Q., Li Y., Guo C., Huang Y., Yu J., Wang W. (2020). *Ginkgo biloba* extract improves brain uptake of ginsenosides by increasing blood-brain barrier permeability via activating A1 adenosine receptor signaling pathway. J. Ethnopharmacol..

[88] Bukowska M., Bogacz A., Wolek M., Mikołajczak P.Ł., Olbromski P., Kamiński A., Czerny B. (2019). Impact of *Curcuma longa* extract on the expression level of brain transporters in *in vivo* model. Herba Pol..

[89] Zhang D.M., Liu H.Y., Xie L., Liu X.D. (2007). Effect of baicalin and berberine on transport of nimodipine on primary-cultured, rat brain microvascular endothelial cells. Acta Pharmacol. Sin..

[90] Li Y., Paxton J.W. (2013). The effects of flavonoids on the ABC transporters: Consequences for the pharmacokinetics of substrate drugs. Expert Opin. Drug Metab. Toxicol..

[91] Mitsunaga Y., Takanaga H., Matsuo H., Naito M., Tsuruo T., Ohtani H., Sawada Y. (2000). Effect of bioflavonoids on vincristine transport across blood-brain barrier. Eur. J. Pharmacol..

[92] Liang G., Li N., Ma L., Qian Z., Sun Y., Shi L., Zhao L. (2016). Effect of quercetin on the transport of ritonavir to the central nervous system in vitro and in vivo. Acta. Pharm..

[93] Reddy D.R., Khurana A., Bale S., Ravirala R., Reddy V.S.S., Mohankumar M., Godugu C. (2016). Natural flavonoids silymarin and quercetin improve the brain distribution of co-administrated P-gp substrate drugs. Springerplus.

[94] He L., Zhao C., Yan M., Zhang L.Y., Xia Y.Z. (2009). Inhibition of P-glycoprotein function by procyanidine on blood-brain barrier. Phytother. Res..

[95] Mahringer A., Karamustafa S., Klotz D., Kahl S., Konkimalla V.B., Wang Y., Wang J., Liu H.Y., Boechzelt H., Hao X. (2010). Inhibition of P-glycoprotein at the blood-brain barrier by phytochemicals derived from traditional Chinese medicine. Cancer Genom. Proteom..

[96] Guimarães L.P.T.P., Rocha G.G., Queiroz R.M., Martins C.A., Takiya C.M., Gattass C.R. (2017). Pomolic acid induces apoptosis and inhibits multidrug resistance protein MRP1 and migration in glioblastoma cells. Oncol. Rep..

[97] Saeed M.E.M., Mahmoud N., Sugimoto Y., Efferth T., Abdel-Aziz H. (2018). Betulinic acid exerts cytotoxic activity against multidrug-resistant tumor cells via targeting autocrine motility factor receptor (AMFR). Front. Pharmacol..

[98] Gupta Y.K., Gupta M., Chaudhary G., Kohli K. (2004). Modulation of antiepileptic effect of phenytoin and carbamazepine by melatonin in mice. Methods Find. Exp. Clin. Pharmacol..

[99] Forcelli P.A., Soper C., Duckles A., Gale K., Kondratyev A. (2013). Melatonin potentiates the anticonvulsant action of phenobarbital in neonatal rats. Epilepsy Res..

[100] Reeta K.H., Mehla J., Pahuja M., Gupta Y.K. (2011). Pharmacokinetic and pharmacodynamic interactions of valproate, phenytoin, phenobarbitone and carbamazepine with curcumin in experimental models of epilepsy in rats. Pharmacol. Biochem. Behav..

[101] Gavzan H., Sayyah M., Sardari S., Babapour V. (2015). Synergistic effect of docosahexaenoic acid on anticonvulsant activity of valproic acid and lamotrigine in animal seizure models. Naunyn Schmiedebergs Arch. Pharmacol..

[102] Salaroglio I.C., Gazzano E., Kopecka J., Chegaev K., Costamagna C., Fruttero R., Guglielmo S., Riganti C. (2018). New tetrahydroisoquinoline derivatives overcome Pgp activity in brain-blood barrier and glioblastoma multiforme in vitro. Molecules.

[103] Rejinold N.S., Yoo J., Jon S., Kim Y.C. (2018). Curcumin as a novel nanocarrier system for doxorubicin delivery to MDR cancer cells: In vitro and in vivo evaluation. ACS Appl. Mater. Interfaces.

[104] Wang L., Li Z., Zhang X., Wang S., Zhu C., Miao J., Chen L., Cui L., Qiao H. (2014). Protective effect of shikonin in experimental ischemic stroke: Attenuated TLR4, p-p38MAPK, NF-κB, TNF-α and MMP-9 expression, up-regulated claudin-5 expression, ameliorated BBB permeability. Neurochem. Res..

[105] Deng G., Ma C., Zhao H., Zhang S., Liu J., Liu F., Chen Z., Chen A.T., Yang X., Avery J. (2019). Anti-edema and antioxidant combination therapy for ischemic stroke via glyburide-loaded betulinic acid nanoparticles. Theranostics.

[106] Zhang Q.L., Fu B.M., Zhang Z.J. (2017). Borneol, a novel agent that improves central nervous system drug delivery by enhancing blood-brain barrier permeability. Drug Deliv..

[107] Tang S., Wang A., Yan X., Chu L., Yang X., Song Y., Sun K., Yu X., Liu R., Wu Z. (2019). Brain-targeted intranasal delivery of dopamine with borneol and lactoferrin co-modified nanoparticles for treating Parkinson’s disease. Drug Deliv..

[108] Zhou J., Wu X., Zhao Z., Wang Z., Li S., Chen C., Yu S., Qu X., Li K., Tian Y. (2020). Preparation of a novel ginkgolide B niosomal composite drug. Open Chem..

[109] Brantley S.J., Argikar A.A., Lin Y.S., Nagar S., Paine M.F. (2014). Herb-drug interactions: Challenges and opportunities for improved predictions. Drug Metab. Dispos..

[110] Han H.K. (2011). Role of transporters in drug interactions. Arch. Pharm. Res..

[111] Lee S.C., Arya V., Yang X., Volpe D.A., Zhang L. (2017). Evaluation of transporters in drug development: Current status and contemporary issues. Adv. Drug Deliv. Rev..

[112] Matsuda A., Karch R., Bauer M., Traxl A., Zeitlinger M., Langer O. (2017). A prediction method for P-glycoprotein-mediated drug-drug interactions at the human blood-brain barrier from blood concentration-time profiles, validated with PET data. J. Pharm. Sci..

[113] Kang S.G., Cho S.E. (2020). Neuroimaging biomarkers for predicting treatment response and recurrence of major depressive disorder. Int. J. Mol. Sci..

[114] Ryu J.Y., Kim H.U., Lee S.Y. (2018). Deep learning improves prediction of drug-drug and drug-food interactions. Proc. Natl. Acad. Sci. USA.

[115] Rohani N., Eslahchi C. (2019). Drug-drug interaction predicting by neural network using integrated similarity. Sci. Rep..

[116] Lee G., Park C., Ahn J. (2019). Novel deep learning model for more accurate prediction of drug-drug interaction effects. BMC Bioinform..

